# An Analysis of Mandibular Characteristics According to Biological Sex Using Three-Dimensional Computed Tomography Scans in Koreans: A Retrospective and Observatoinal Study

**DOI:** 10.3390/medicina62020398

**Published:** 2026-02-19

**Authors:** Byeongjun Kim, Junghyun Lee, Donghyun Lee, Kuylhee Kim, Jiwon Jeong, Soyeon Jung

**Affiliations:** 1 Department of Plastic and Reconstructive Surgery, Dongtan Sacred Heart Hospital, Hallym University College of Medicine, Hwaseong 18450, Republic of Korea; echo0118@gmail.com (B.K.); jhyun5221@gmail.com (J.L.); 2 Department of Plastic and Reconstructive Surgery, Hallym University Kangdong Sacred Heart Hospital, Seoul 05355, Republic of Korea; dh2120@naver.com (D.L.); drkhkimbb@gmail.com (K.K.); swjej1234@gmail.com (J.J.)

**Keywords:** mandible, sex, sexual dimorphism, facial bones

## Abstract

*Background and Objectives*: With the increasing demand for gender-affirming procedures, facial feminization surgery (FFS) has become an essential component in the management of patients with gender dysphoria. In this study, ‘male’ and ‘female’ refer to biological sex as recorded in the medical record; gender identity was not assessed. The mandible is widely recognized as one of the most sexually dimorphic facial bones and plays a critical role in defining masculine and feminine facial contours. However, quantitative mandibular data directly applicable to surgical planning for FFS, particularly in Asian populations, remain limited. The purpose of this study was to analyze gender differences in mandibular morphology using three-dimensional (3D) computed tomography (CT) images and to provide clinically relevant anatomic data applicable to mandibular contouring in FFS. *Materials and Methods*: In this single-center retrospective study, 275 Korean patients who underwent facial CT between January 2017 and December 2019 were enrolled. Three-dimensional cephalometric analysis was performed to obtain surgically relevant mandibular measurements, including angular, linear, and transverse parameters, as well as non-metric characteristics such as chin shape and inferior mandibular border contour. Statistical comparisons were conducted to evaluate gender differences. *Results*: Significant gender differences were observed in mandibular angle (*p* < 0.001), mandible length (*p* < 0.001), antegonial notch distance (*p* < 0.001), intercondylar width (*p* < 0.001), and intergonial width (*p* < 0.001). Ramus length and chin width did not demonstrate statistically significant differences. Non-metric analysis revealed significant gender differences in chin morphology and inferior mandibular border contour (*p* < 0.01). Males predominantly exhibited a round or square chin (79.5%) and a rocker-shaped inferior border, whereas females commonly demonstrated a pointed chin (82.3%) and a straight inferior mandibular border (94.4%). *Conclusions*: The sexual dimorphism of the mandible in the Korean population is characterized by differences in angularity, transverse width, antegonial morphology, and inferior border contour. These findings provide population-specific morphological reference ranges that may support individualized preoperative assessment for mandibular contouring in facial feminization surgery.

## 1. Introduction

Differences in facial skeletal characteristics based on biological sex have become increasingly important with the growing demand for facial feminization and masculinization procedures. Although facial feminization surgery (FFS) is performed in the context of gender-affirming care, the underlying skeletal modifications are based on morphological differences associated with biological sex rather than gender identity itself. Although the sexual dimorphism of the facial skeleton has been extensively studied in anthropology, osteology, and forensic science, most of the existing literature focuses on sex estimation rather than surgical applicability [1,2].

Among facial bones, the mandible exhibits pronounced sexual dimorphism and plays a pivotal role in determining lower facial width, angularity, and overall facial contour. From a surgical perspective, the mandible is particularly relevant because its size, angular configuration, and inferior border morphology are directly modifiable through contouring osteotomy and genioplasty. Additionally, the mandible is the most durable facial bone and retains its morphology better than other craniofacial structures, making it a reliable substrate for morphological analysis [1,2].

Metric analysis of craniofacial structures has traditionally relied on two-dimensional cephalometric techniques. Conventional cephalometry, first introduced by Pacini in 1922, has been widely used to evaluate facial proportions and skeletal relationships [3]. However, two-dimensional cephalometry is inherently limited by projection errors, rotational distortion, and reduced accuracy in patients with facial asymmetry, restricting its usefulness in detailed surgical planning [4,5].

With advances in imaging technology, three-dimensional CT-based cephalometric analysis has become increasingly important for preoperative evaluation and surgical simulation in craniofacial surgery. Three-dimensional imaging allows for the accurate assessment of mandibular morphology in all spatial dimensions and provides data that can be directly translated into surgical planning, particularly in complex procedures such as facial feminization surgery [6].

Despite the clinical relevance of mandibular morphology in FFS, population-specific 3D morphological data applicable to surgical planning remain scarce in Korean individuals. Given the increasing demand for gender-affirming surgery in Asian populations, there is a critical need for quantitative mandibular data that reflect ethnic and anatomic specificity rather than extrapolation from Western datasets. Accordingly, the novelty of the present study lies in its deliberate alignment of three-dimensional mandibular morphometric analysis with surgically relevant regions and features commonly addressed during facial feminization surgery, rather than in a descriptive characterization of sexual dimorphism alone. Accordingly, the present analysis focuses on mandibular dimensions and features that are directly relevant to surgical contouring, as these regions are commonly addressed during mandibular osteotomy and genioplasty and play a key role in determining lower facial width and contour.

The purpose of this study was to analyze biological sex-based differences in mandibular morphology in a Korean population using three-dimensional CT-based measurements and non-metric characteristics and to provide population-specific anatomic reference data applicable to preoperative planning in facial feminization surgery. By identifying surgically relevant morphological differences between males and females, this study aims to provide an anatomic framework applicable to preoperative planning in facial feminization surgery.

## 2. Materials and Methods

In this retrospective study, we performed a cephalometric analysis of 3D-CT imaging data collected in a single center over a 3-year period. Subjects were selected from patients who underwent facial CT between January 2017 and December 2019 and satisfied the following inclusion criteria: (1) Korean adult men or women aged older than 15 years (because mandibular growth becomes stable at that age), (2) patients with no congenital or acquired dentofacial deformities (e.g., cleft lip or palate, craniofacial syndrome, jaw protrusion or retrusion, or post-traumatic deformity), (3) patients with no obvious facial asymmetry, (4) patients with no history of oral and maxillofacial surgery, (5) patients with a class I occlusal relationship, (6) patients without an edentulous mandible, (7) patients with no history of dental implant, and (8) patients with no mandible fracture. This study was designed and conducted following the Strengthening the Reporting of Observational Studies in Epidemiology (STROBE) guidelines.

Age stratification or age-adjusted multivariable analysis was not performed due to the study’s retrospective design and uneven age distribution; therefore, age-related effects were considered during interpretation rather than controlled analytically. Patients with clinically apparent facial asymmetry were excluded to reduce measurement noise and improve landmarking consistency for sex-based morphometric comparisons; however, this may limit generalizability to real-world facial feminization surgery populations where asymmetry is common. In the current study, we enrolled 275 Korean individuals (n = 275), comprising 151 men and 124 women, whose mean age was 42.40 ± 17.40 years (range, 16–87 years). The current study was approved by the Institutional Review Board (IRB) of our medical institution. All CT scans were obtained for clinical diagnostic purposes unrelated to facial feminization surgery. As this was a retrospective study based on clinically acquired imaging, the study population may not fully represent the general population. However, strict exclusion criteria were applied to minimize the influence of craniofacial pathology or deformity on mandibular morphology. The most common indications for facial CT at our institution include trauma evaluation, inflammatory/infectious conditions, and oncological or preoperative diagnostic work-up; CT indication was not used as a selection variable in this study.

**Figure 1:**
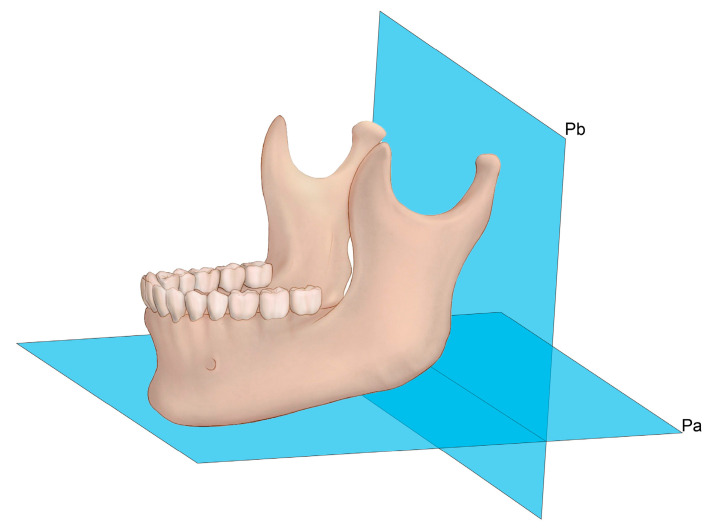
The reference planes of the mandible. A three-dimensional illustration of the mandible showing the reference planes used for morphometric measurements. Pa: The bottom plane of the mandibular corpus. Pb: The posterior plane of the mandibular ramus.

**Figure 2:**
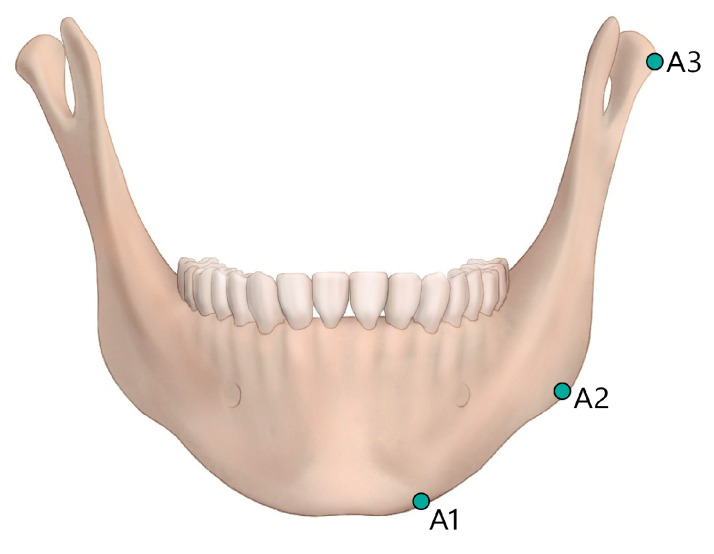
Frontal view of mandibular landmarks. A frontal view of the mandible showing the bilateral anatomic landmarks used for transverse measurements. A1: Mental tubercle. A2: The most lateral point of the right gonion. A3: The most lateral point of the right condylar head.

**Figure 3:**
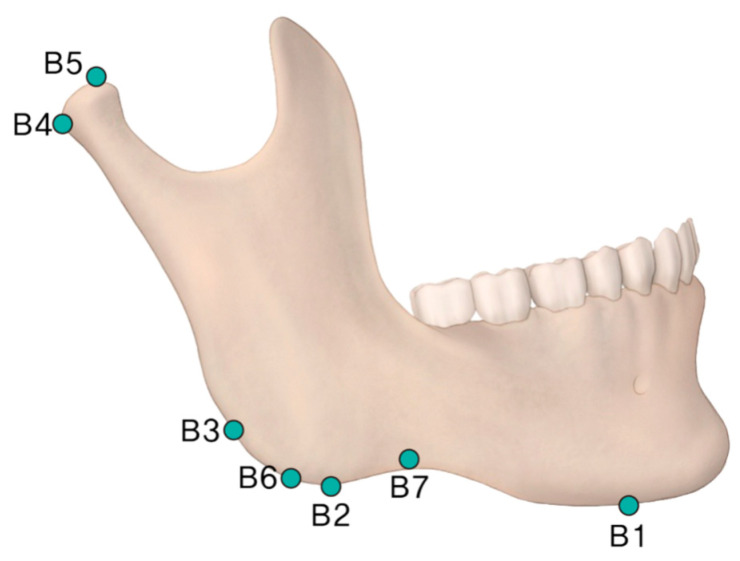
Mandibular landmarks used for angular measurements. Anatomic landmarks (B1–B7) are defined in Table 1. A lateral view of the mandible illustrating the anatomic landmarks used for morphometric analysis. B1: The anteroinferior point of the mandibular corpus. B2: The posteroinferior point of the mandibular corpus. B3: The posterior protruding point of the mandibular corpus. B4: The posterior point of the mandibular condyle. B5: The highest point of the mandibular condyle. B6: The most inferior point of the gonion. B7: The most concave part of the lower border of the mandible.

**Figure 4:**
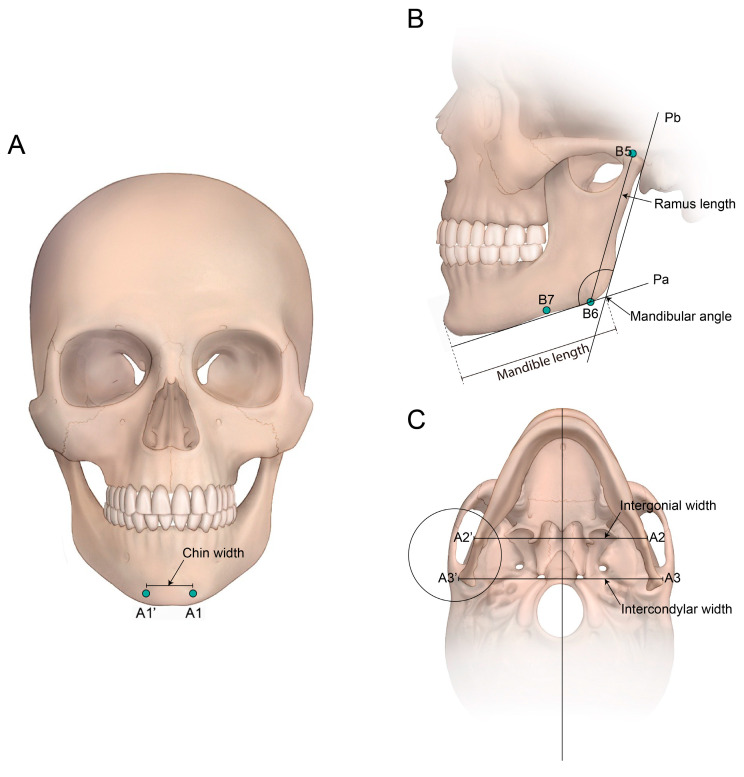
Linear and angular measurements of the mandible. (**A**) A frontal view illustrating chin width, defined as the distance between the right and left mental tubercles (A1-A1′). (**B**) A lateral view demonstrating the mandibular angle, formed by the intersection of the bottom plane of the mandibular corpus (Pa) and the posterior plane of the mandibular ramus (Pb). Mandible length was defined as the distance from the most anterior point of the chin to a line placed along the posterior border of the ramus, and ramus length was defined as the distance between B5 and B6. (**C**) An inferior view showing transverse measurements, including intergonial width (A2-A2′) and intercondylar width (A3-A3′).

Each patient’s head was placed in the CT scanner (Siemenx, Erlangen, Germany) on a foam platform with the Frankfort horizontal plane parallel to the floor. Then, their head was placed in the center of the CT scanner in line with the midsagittal plane. The obtained images were sent to the image viewer, PiView STAR (Infinitt Co., Ltd., Seoul, Republic of Korea), which was used to identify conventional cephalometric hard tissue landmarks and to measure the distance and the angle in each dimension (Figure 1, Figure 2, Figure 3 and Figure 4). Measurements were taken by two independent researchers in order to minimize the identification error in selecting the landmarks and calculating measurements. Intraclass coefficient (ICC) analysis was performed and interpreted as the Cicchetti categorization system. The Cicchetti categorization system classifies the ICC as follows: <0.40, poor agreement; 0.40–0.59, fair agreement; 0.60–0.74, good agreement; and 0.75–1, excellent agreement [7]. The detailed definitions of the landmarks used are provided in Table 1. In addition, the detailed measurements of distances and angle are defined in Table 2. Mandible length was intentionally defined using the anterior chin point to the posterior ramus reference line (Pb), rather than the conventional Go–Gn distance, to improve reproducibility in 3D datasets where gonion localization may vary with ramus flaring and mandibular angle morphology. Sagittal mandibular measurements were performed exclusively on the left side to reduce variability related to facial asymmetry and to ensure measurement consistency across subjects in this retrospective dataset.

**Figure 5:**
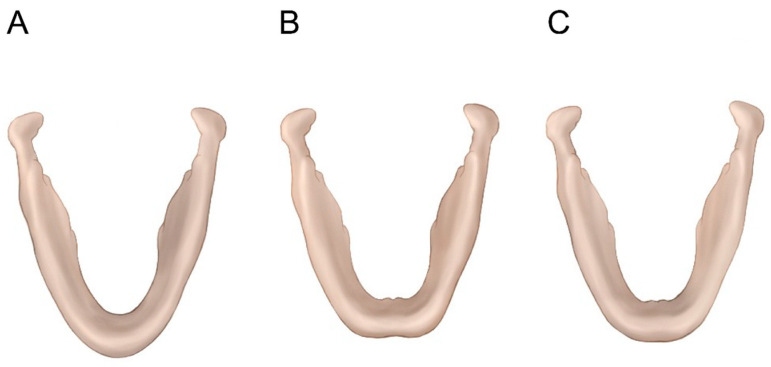
Classification of chin shape. An inferior view of the mandible illustrating the classification of chin morphology. (**A**) Pointed shape. (**B**) Square shape. (**C**) Round shape.

**Figure 6:**
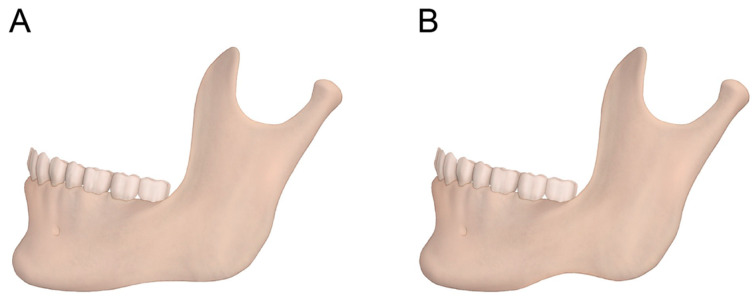
Classification of the lower border of the mandible. A lateral view of the mandible illustrating the classification of the lower border morphology. (**A**) The contour with straight line of the inferior mandibular margin. (**B**) The contour with notch of the inferior mandibular margin.

Non-metric characteristics, including chin shape and inferior mandibular border contour, were assessed using three-dimensional CT images. These features were independently classified by two observers who were blinded to patient biological sex. Prior to evaluation, observer calibration was performed using representative cases to standardize classification criteria. Interobserver agreement was assessed, and any discrepancies were resolved by consensus, to enhance the reproducibility of these categorical classifications. The shape of the chin and the contour of the lower border of the mandible were recorded using 3D-CT imaging. The shape of the chin was classified as pointed, square, or round (Figure 5). The lower border of the mandible was classified as rocker-shaped or straight (Figure 6).

These measurements were averaged for statistical analysis. The independent *t*-test and Pearson’s chi-squared test were, respectively, used to detect differences in measurements or non-metric characteristics of the mandible between males and females. Statistical significance was classified as a *p*-value < 0.05. The Statistical Product and Service Solution 27.0 software (SPSS Inc., Chicago, IL, USA) was used for statistical calculation.

## 3. Results

The studied population (n = 275) consisted of 54.9% males and 45.1% females with no significant differences in age. ICC values for interobserver variation are presented in Table 3. The interobserver variation in external measurements showed excellent results for all measurements according to the Cicchetti categorization system (0.921 < ICC < 0.996).

Significant differences between males and females were found for five of seven 3D-CT-based mandibular measurements, excluding ramus length and chin width. First, the frontal measurements revealed that chin width was not significantly different between males and females (male, 22.7 ± 4.3; female, 22.1 ± 3.7; *p* = 0.703).

The sagittal measurements of the left side showed that females (123.3 ± 3.7) have a greater mean mandibular angle compared to males (120.7 ± 4.0), reaching statistical significance (*p* < 0.001). Mandible length (male, 73.5 ± 2.3; female, 68.6 ± 4.2; *p* < 0.001) and antegonial notch distance (male, 2.6 ± 0.7; female, 1.2 ± 0.3; *p* < 0.001) were significantly different between males and females. The ramus length of male patients (58.3 ± 4.8) was longer than that of female patients (56.1 ± 2.6) without any significant difference (*p* = 0.073).

The transverse measurements revealed that intercondylar width (male, 126.9 ± 7.6; female, 122.6 ± 6.1; *p* < 0.001) and intergonial width (male, 101.3 ± 4.5; female, 98.4 ± 2.2; *p* < 0.001) were significantly different between males and females.

The shape of the chin and the lower border of the mandible were significantly different between males and females (*p* < 0.01). First, the chin of males was mainly round or square (79.5%), whereas the chin of females was mainly pointed (82.3%) (Table 3). Additionally, most males (67.5%) had a notched lower border of the mandible, whereas most females (94.4%) had a straight lower border (Table 4). In addition to statistical significance, the magnitude of sex differences was largest for antegonial notch distance and mandible length (large standardized effects), followed by transverse widths and mandibular angle (moderate effects), whereas chin width showed a small effect.

## 4. Discussion

Mandible measurements using 3D imaging and cephalometry are both valuable tools for evaluating craniofacial structures, but they serve different purposes and have their own advantages and limitations. Cephalometry is a two-dimensional X-ray imaging technique that provides a detailed assessment of the skeletal and soft tissue structures of the head and face and is widely used in orthodontics to diagnose and plan treatment for dental and skeletal malocclusions. Cephalometric analysis can provide measurements of angles and linear distances to assess the positional relationships of the maxilla, mandible, and other facial structures. However, from a surgical perspective, conventional two-dimensional cephalometry has inherent limitations in accurately representing complex three-dimensional mandibular morphology. On the other hand, three-dimensional imaging techniques such as computed tomography allow for the generation of highly detailed 3D models of the craniofacial skeleton, enabling a comprehensive assessment of skeletal structures in all spatial dimensions. This characteristic is particularly advantageous in procedures that require precise preoperative planning, such as facial feminization surgery (FFS) [6].

There are known limitations to cephalometry, including projective displacement, rotational errors, and linear projective transformation, which reduce the reliability and reproducibility of measurements [4,8]. Moreover, most cephalometric analyses are susceptible to distortion in the presence of facial asymmetry, which is not uncommon in clinical practice [5]. Given these limitations, three-dimensional imaging is increasingly preferred when a detailed morphological evaluation of the mandible is required for surgical decision making. Consequently, we elected to use 3D-CT data to measure mandibular length, angularity, and transverse dimensions in Korean individuals.

In the present study, mandibular length was defined as the distance from the most anterior point of the chin to a line placed along the posterior border of the mandibular ramus, rather than the conventional gonion–gnathion (Go–Gn) distance commonly used in cephalometric analyses [9]. This methodological choice was based on considerations of objectivity and reproducibility in three-dimensional assessment. The gonion represents a geometric construct influenced by mandibular angle morphology and ramus flaring, and its precise localization can be challenging, even in three-dimensional datasets. In contrast, the posterior plane of the mandibular ramus (Pb) provides a more consistent and anatomically well-defined reference surface, while the most anterior point of the chin can be reliably identified across subjects.

It should be noted that the traditional Go–Gn measurement represents an oblique linear distance and therefore may yield slightly longer absolute values compared with projection-based measurements such as those used in the present study. Nevertheless, previous investigations have shown that differences between these measurement approaches do not substantially alter overall morphological trends or patterns of sexual dimorphism. Accordingly, although the absolute mandibular length values in our study may appear shorter than those reported in studies using the Go–Gn distance, the observed gender-related differences and directional tendencies remain comparable.

With regards to the evaluation of antegonial morphology, previous studies have frequently quantified the antegonial notch using area-based measurements. Although such approaches may capture the overall shape of the notch, they are inherently sensitive to landmark selection and contour tracing, which can introduce subjectivity and reduce reproducibility. In the present study, the antegonial notch was therefore assessed using a linear metric, defined as the perpendicular distance between the most concave point of the lower border of the mandible (B7) and the mandibular plane (Pa).

This measurement strategy was adopted to enhance objectivity and consistency by relying on clearly defined anatomic landmarks and reference planes rather than contour-dependent area calculations. A similar line-based approach has been employed in previous three-dimensional morphological studies, including that of Schutz et al., supporting the validity of this method for evaluating antegonial morphology [10]. By focusing on the depth of the antegonial notch relative to a standardized mandibular plane, this linear measurement provides a practical and reproducible parameter that is well suited for three-dimensional CT-based analysis and surgical planning.

Previous studies across different populations have consistently demonstrated the sexual dimorphism of the mandible, although the degree and specific parameters vary by ethnicity. In an Indian population aged 18–30 years, analysis of 304 digital cephalometric radiographs revealed that females exhibited significantly larger gonial angles than males (*p* = 0.035) [11]. In a Korean population study conducted at Seoul St. Mary’s Hospital, Lin et al. analyzed 240 cranial CT scans and reported that 8 of 11 variables related to mandibular ramus flexure showed significant sexual dimorphism, with upper ramus vertical height demonstrating the greatest difference (77.1%) [12]. In an Israeli population, Tunis et al. evaluated 25 CT-based mandibular measurements and found significant gender differences in most linear, angular, and transverse parameters, including mandibular length, ramus length, mandibular angle, bicondylar breadth, and bigonial breadth [13].

In the present study, five of seven mandibular measurements demonstrated statistically significant gender differences using 3D-CT imaging. Overall, our findings are consistent with previously published CT-based studies, supporting the presence of robust sexual dimorphism in mandibular morphology. However, chin width and ramus length did not show statistically significant differences between males and females in our cohort. The lack of statistical significance for ramus length and chin width may reflect population-specific similarity in these dimensions, but it may also be influenced by variability in landmark identification and the wide age distribution, which can increase within-group variance and reduce power for certain parameters. Clinically, these findings suggest that not all lower-face components require uniform modification in feminization planning; rather, transverse width, mandibular angle configuration, and inferior border contour may provide more discriminative reference features in this cohort.

It should be noted that the relative effect size categories reported in this study reflect standardized statistical magnitude rather than direct clinical or surgical priority. Although mandible length demonstrated a large effect size, this parameter represents a global morphometric characteristic, whereas features such as mandibular angle, transverse width, and inferior border contour are more closely associated with regions typically emphasized during mandibular contour assessment in facial feminization surgery. Accordingly, statistical effect size and clinical interpretability should be regarded as distinct conceptual dimensions. This discrepancy suggests that not all mandibular dimensions contribute equally to sexual dimorphism and highlights the importance of population-specific analysis when defining surgically relevant anatomic targets. Another important limitation of this study is the wide age range of the included subjects. Although mandibular growth is generally considered complete after adolescence, age-related skeletal remodeling, including changes in mandibular angle, inferior border morphology, and cortical bone characteristics, may influence mandibular shape in older individuals. Because age-stratified or age-adjusted analyses were not performed, the observed morphological differences may partially reflect age-related variation in addition to biological sex–based dimorphism.

In addition to metric analysis, non-metric characteristics of the mandible were evaluated. Hu et al. reported that rocker-shaped mandibular bases predominated in males, whereas straight inferior borders were more common in females in a Korean population [14]. Similarly, Nagaraj et al. found that all male mandibles exhibited a rocker-shaped inferior border, whereas all female mandibles demonstrated a straight inferior border in a South Indian population [15]. In contrast, Deana and Alves reported less pronounced sexual dimorphism of the mandibular base in a Brazilian population, with rocker-shaped borders observed in both sexes [16]. In our study, a rocker-shaped inferior mandibular border was significantly more common in males (67.5%), whereas a straight inferior border predominated in females (97.4%), closely aligning with previous findings in Korean and South Indian populations [14,15].

Chin morphology also demonstrated significant sexual dimorphism. Hu et al. reported that square chins were more common in males, whereas pointed chins predominated in females in a Korean population [14]. Similar findings were reported in South Indian and Brazilian populations, where males more frequently exhibited square or bilobate chins and females more frequently exhibited pointed chins [15,16]. Consistent with these studies, our results showed that round or square chins predominated in males (79.5%), whereas pointed chins were most common in females (82.3%). These non-metric characteristics provide clinically intuitive information that complements quantitative measurements. However, their classification inherently involves a degree of subjectivity. Accordingly, these findings should be interpreted as supportive descriptive features rather than definitive criteria for surgical decision making. Although formal kappa statistics were not calculated for non-metric classifications, these features were intended as supportive descriptive findings and should be interpreted accordingly.

Measurements and non-metric characteristics of the mandible are important components of preoperative planning for facial feminization surgery. The mandible is a prominent determinant of lower facial width and contour prominence, and male mandibles are typically larger and more contour-prominent than female mandibles [17]. Based on our findings, a relatively obtuse mandibular angle, reduced transverse width, straighter inferior border, and pointed chin morphology may serve as morphological features associated with a feminine mandibular contour in the Korean population. Rather than defining prescriptive surgical targets, the present findings should be regarded as population-specific anatomic reference data that may assist surgeons in contextualizing biological sex-based mandibular morphology during preoperative planning.

This study has several limitations. First, a single-detector CT system was used rather than a multi-detector CT, which may affect image resolution. Second, the retrospective study design limits control over potential confounding variables and may introduce selection bias. Lastly, sagittal measurements were limited to the left side of the mandible to minimize the confounding effects of asymmetry. Previous studies have reported statistically significant bilateral mandibular asymmetry, and future investigations incorporating bilateral measurements and postoperative outcomes are warranted [18]. In summary, this study provides three-dimensional CT-based reference data describing the sexual dimorphism of the mandible in a Korean population. While significant differences were observed in selected mandibular parameters, these findings should be interpreted within the context of the study’s limitations, including the wide age range, unilateral sagittal assessment, and the descriptive nature of non-metric classifications. Accordingly, the present results are intended to support contextual understanding of sex-based mandibular morphology rather than to serve as prescriptive surgical guidelines.

## 5. Conclusions

This study demonstrates that the biological sex-based dimorphism of the mandible in Korean individuals is characterized by differences in mandibular angularity, transverse width, antegonial morphology, and inferior border contour. These findings provide population-specific anatomic reference information derived from three-dimensional CT analysis. Rather than serving as prescriptive surgical guidelines, the results may assist surgeons in understanding typical morphological patterns during preoperative assessment for facial feminization surgery. Given the retrospective design, unilateral sagittal measurements, and wide age range of the cohort, the findings should be interpreted within these limitations and should not be generalized uncritically to other populations. Future studies incorporating age-adjusted analyses, bilateral assessment, and correlation with surgical outcomes are warranted.

## Figures and Tables

**Table 1:** Descriptions of landmarks used to derive mandibular morphometric measurements.

Landmarks and Planes (Left)	
Bilateral Landmarks (Right)	
A1 (A1′)	Mental turbercles
A2 (A2′)	the most lateral points of the right and left gonions
A3 (A3′)	the most lateral points of the right and left condyle heads
B1 (B1′)	Anteroinferior point of mandibular corpus
B2 (B2′)	Posteroinferior point of mandibular corpus
B3 (B3′)	Posterior protruding point of mandibular corpus
B4 (B4′)	Posterior point of mandibular condyle
B5 (B5′)	Highest point of mandibular condyle
B6 (B6′)	The most inferior point of the gonion
B7 (B7′)	The most concave part of the lower border of mandible
Pa	The bottom plane of mandibular corpus
Pb	The posterior plane of mandibular ramus

**Table 2:** Definitions of CT-based mandibular measurements.

Measurement	Definition
Ramus length	The distance from the highest point on the condyle (B5) to the gonion (B6)
Mandible length	The distance from the most anterior point of the chin to a line placed along the posterior border of the ramus
Mandibular angle	The angle formed by the inferior border of the mandibular body (Pa) and the posterior border of the ramus (Pb)
Intercondylar width	The distance between the most lateral points of the right and left condyle heads (A3-A3′)
Intergonial width	The distance between the right and left gonions (A2-A2′)
Chin width	The distance between the right and left mental tubercles (A1-A1′)
Antegonial notch distance	The distance between the most concave part of the lower border of the mandible (B7) and the mandibular plane line (Pa)

All sagittal linear and angular measurements were recorded on the left side.

**Table 3:** Interobserver reliability tests: intraclass correlation coefficient (ICC) analysis.

Measurement	Interobserver Variation [Mean (SD)]		
	First Observer	Second Observer	ICC
Intercondylar width	123.44 (±8.31)	123.99 (±8.23)	0.996
Intergonial width	99.62 (±6.16)	99.96 (±6.25)	0.995
Mandibular angle	120.94 (±9.50)	121.97 (±8.58)	0.952
Mandible length	72.42 (±7.61)	72.81 (±8.12)	0.965
Ramus length	57.47 (±5.59)	56.53 (±6.63)	0.921
Chin width	24.16 (±7.53)	24.51 (±7.83)	0.983
Antegonial notch distance	2.05 (±1.31)	2.39 (±1.02)	0.978

**Table 4:** Statistical analysis of CT-based mandibular measurements between male and female.

Measurements Number		Male	Female	*p*-Value ^(a), (b)^
		151	124	
Intercondylar width		126.9 ± 7.6	122.6 ± 6.1	<0.001
Intergonial width		101.3 ± 4.5	98.4 ± 2.2	<0.001
Mandibular angle		120.7 ± 3.7	123.3 ± 4.0	<0.001
Mandible length		73.5 ± 2.3	68.6 ± 4.2	<0.001
Ramus length		58.3 ± 4.8	56.1 ± 2.6	0.073
Chin width		22.7 ± 4.3	22.1 ± 3.7	0.703
Antegonial notch distance		2.6 ± 0.7	1.2 ± 0.3	<0.001
Chin shape	Pointed	31	102	<0.001
	Round/Square	120	22	
Lower border	Notch	102	7	<0.001
	Straight	49	117	

^(a)^ *t*-test; *p* < 0.05, statistically significant, ^(b)^ Chi square test; *p* < 0.05, statistically significant.

## Data Availability

Data are contained within the article.

## Abbreviations

CT, computed tomographs; 3D, three-dimensional; FFS, facial feminization surgery.

## References

[1] Vinay G., Mangala Gowri S.R., Anbalagan J. (2013). Sex determination of human mandible using metrical parameters. J. Clin. Diagn. Res..

[2] Giles E. (1964). Sex determination by discriminant function analysis of the mandible. Am. J. Phys. Anthropol..

[3] Shaw R.B., Kahn D.M. (2007). Aging of the midface bony elements: A three-dimensional computed tomographic study. Plast. Reconstr. Surg..

[4] Kapila S., Conley R.S., Harrell W.E. (2011). The current status of cone beam computed tomography imaging in orthodontics. Dentomaxillofac. Radiol..

[5] Xia J.J., Gateno J., Teichgraeber J.F. (2009). New clinical protocol to evaluate craniomaxillofacial deformity and plan surgical correction. J. Oral Maxillofac. Surg..

[6] Dang B.N., Hu A.C., Bertrand A.A., Chan C.H., Jain N.S., Pfaff M.J., Lee J.C., Lee J.C. (2022). Evaluation and treatment of facial feminization surgery: Part II. lips, midface, mandible, chin, and laryngeal prominence. Arch. Plast. Surg..

[7] Cicchetti D.V. (1994). Guidelines, criteria, and rules of thumb for evaluating normed and standardized assessment instruments in psychology. Psychol. Assess..

[8] Lin H.S., Li J.D., Chen Y.J., Lin C.C., Lu T.W., Chen M.H. (2014). Comparison of measurements of mandible growth using cone beam computed tomography and its synthesized cephalograms. BioMed. Eng. OnLine.

[9] Agurto-sanhueza P., Roco K., Navarro P., Neyem A., Sumonte N.I., Ottone N.E. (2025). Simplified diagnosis of mandibular asymmetry in panoramic radiographs through digital processing and its prospective integration with artificial intelligence: A pilot study. Appl. Sci..

[10] Schutz C., Denes B.J., Kiliaridis S., Antonarakis G.S. (2022). Mandibular antegonial notch depth in postpubertal individuals: A longitudinal cohort study. Clin. Exp. Dent. Res..

[11] Belaldavar C., Acharya A.B., Angadi P. (2019). Sex estimation in indians by digital analysis of the gonial angle on lateral cephalographs. J. Forensic Odonto-Stomatol..

[12] Lin C., Jiao B., Liu S., Guan F., Chung N.E., Han S.H., Lee U.-Y. (2014). Sex determination from the mandibular ramus flexure of Koreans by discrimination function analysis using three-dimensional mandible models. Forensic Sci. Int..

[13] Tunis T.S., Sarig R., Cohen H., Medlej B., Peled N., May H. (2017). Sex estimation using computed tomography of the mandible. Int. J. Legal Med..

[14] Hu K.S., Koh K.S., Han S.H., Shin K.J., Kim H.J. (2006). Sex determination using nonmetric characteristics of the mandible in Koreans. J. Forensic Sci..

[15] Nagaraj T., Veerabasvaiah B.T., James L., Dev Goswami R., Narayanan S., Keerthi I. (2016). Use of non-metric characteristics of mandible in sex determination. J. Med. Radiol. Pathol. Surg..

[16] Deana N.F., Alves N. (2017). Nonmetrical sexual dimorphism in mandibles of Brazilian individuals. Biomed. Res..

[17] Callen A.L., Badiee R.K., Phelps A., Potigailo V., Wang E., Lee S., Talbott J., Glastonbury C., Pomerantz J.H., Narvid J. (2021). Facial Feminization Surgery: Key CT Findings for Preoperative Planning and Postoperative Evaluation. Am. J. Roentgenol..

[18] Kim Y.H., Kang S.J., Sun H. (2016). Cephalometric Angular Measurements of the Mandible Using Three-Dimensional Computed Tomography Scans in Koreans. Arch. Plast. Surg..

